# Multi-ontology embeddings approach on human-aligned multi-ontologies representation for gene-disease associations prediction^☆^

**DOI:** 10.1016/j.heliyon.2023.e21502

**Published:** 2023-10-30

**Authors:** Yihao Wang, Philipp Wegner, Daniel Domingo-Fernández, Alpha Tom Kodamullil

**Affiliations:** a Department of Bioinformatics, Fraunhofer Institute for Algorithms and Scientific Computing (SCAI), Sankt Augustin, 53757, Germany; b Rheinische Friedrich-Wilhelms-Universität Bonn, Bonn, 53115, Germany; c German Center for Neurodegenerative Diseases (DZNE), Bonn, 53127, Germany

**Keywords:** Multi-ontology, Natural language processing

## Abstract

**Objectives:**

Knowledge graphs and ontologies in the biomedical domain provide rich contextual knowledge for a variety of challenges. Employing that for knowledge-driven NLP tasks such as gene-disease association prediction represents a promising way to increase the predictive power of a model.

**Methods:**

We investigated the power of infusing the embedding of two aligned ontologies as prior knowledge to the NLP models. We evaluated the performance of different models on some large-scale gene-disease association datasets and compared it with a model without incorporating contextualized knowledge (BERT).

**Results:**

The experiments demonstrated that the knowledge-infused model slightly outperforms BERT by creating a small number of bridges. Thus, indicating that incorporating cross-references across ontologies can enhance the performance of base models without the need for more complex and costly training. However, further research is needed to explore the generalizability of the model. We expected that adding more bridges would bring further improvement based on the trend we observed in the experiments. In addition, the use of state-of-the-art knowledge graph embedding methods on a joint graph from connecting OGG and DOID with bridges also yielded promising results.

**Conclusion:**

Our work shows that allowing language models to leverage structured knowledge from ontologies does come with clear advantages in the performance. Besides, the annotation stage brought out in this paper is constrained in reasonable complexity.

## Graphical abstract

This Graphical abstract shows the main workflow of our approach. First, we used bridges to manually align two ontologies. Then we use some SOTA Embedding method, and feed the embedding and dataset(Gene-disease-association) together to further fine tune the nlp model.

**Figure:**
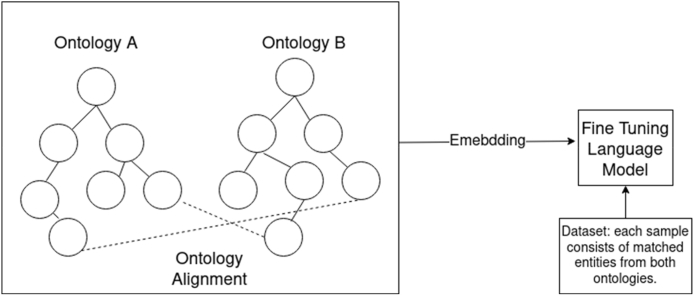

## 1 Introduction

Gene-disease association prediction is a widely researched topic in the biomedical domain. Predicting unseen gene-disease pairs more accurately or exploring further undiscovered associations can help people gain a better understanding of the genetic basis of diseases.

Over the past few years, with the success of neural networks in natural language processing problems, researchers have been increasingly attempting to apply these methods to predict gene-disease associations [1,2].

Much biomedical knowledge can be encoded using ontologies, which come with a hierarchical structure of concepts. Some examples of widely-used ontologies are the Human Disease Ontology (DOID) [3], Gene Ontology (GO) [4], and Ontology of Genes and Genomes (OGG) [5]. In order to derive a numerical representation of the concepts that machine-learning algorithms can process, the study of ontology embedding is essential. An approach adopted in this work considers the underlying Knowledge Graph (KG) derived from the ontology and applies well-established knowledge graph embedding strategies (KGE) to it. Common KGE approaches are translational distance models: TransE [6], TransR [7], bilinear model: RESCAL [8], DistMult [9], or neural network-based models [10]. Other methods, such as Onto2Vec [11] and OPA2Vec [12], are ontology-specific embedding strategies. Recently, OWL2VEC* [13], an ontology embedding method that considers the graph structure, lexical information and logical constructor, delivers a promising result in ontology embeddings.

Deep learning has made significant breakthroughs in natural language understanding tasks in the last decade [14,15]. ERNIE [16], a novel approach to enhancing language representation with informative entities, can be considered an extension of BERT. Additional knowledge about the entities of the input sequence, i.e., the knowledge from a graph structure data that includes the entity, may improve the model's performance on various down-streaming tasks.

In this paper, we chose the OGG and DOID ontologies for the prediction task of gene-disease associations. Next, we manually built bridges (cross-references) between the two ontologies in the preprocessing stage. Subsequently, the resulting joint ontology can be now embedded, and the resulting numerical knowledge representation can be fed as contextual knowledge to the knowledge-infused model ERNIE [16]. In the following, we elaborate on how this method can improve the performance in the gene-disease association prediction task compared to a BERT model [15] at baseline. Lastly, following a similar approach introduced by Nunes et al. [1], we explore the performance of state-of-the-art KGE methods for embedding the context knowledge.

## 2 Methods

### 2.1 Dataset and ontologies

To demonstrate our approach, we used TBGA [17], a large-scale dataset of gene-disease associations, to benchmark our method on the gene-disease association prediction task. TBGA is a natural language text corpus, where each sentence contains one gene and one disease. While in the original dataset, a gene-disease pair has four different relations, i.e., “NA (Not associated)” “therapeutic” “Biomarker” and “Genomic” [17]. The non-relation (NA) class dominated the dataset, and imbalanced samples might cause a long tail effect, so we have tried to balance the dataset. Furthermore, we considered a slightly relaxed version of the problem by combining the three associated classes together and balanced the dataset again (i.e., association and non-association examples are approximately 50 %) **(**Supplementary Table 1**)** for a binary classification.

Additionally, we employed a second dataset, DisGeNET, to explore the generalization capabilities of our attempt. DisGeNet is a gold standard dataset for gene-disease associations created using a text-mining approach on different expert-curated databases [18]. Since DisGeNET contains over 100,000 gene-disease pairs of existing and potential association pairs [18], we randomly chose 5 % of the data as positive samples and created an equal number of negative samples in order to have a dataset of a similar size as TBGA. The randomness of negative samples was constrained by the dataset itself, all negative samples have never shown as associated pairs in the dataset. These could still be false negatives regarding the original dataset because not every gene-disease association is covered by DisGeNET.

Since the dataset was presented only in gene-disease pairs, to make the dataset match the data style of TBGA for the natural language classification task, we performed prompt engineering on DisGeNET with the simplest arrangement, which is “Is DISEASE related to GENE?”.

For ontologies that provided information for entities, we employed the DOID and OGG. In some related works [1], Gene Ontology (GO) is a widely-used ontology in the field of genes, which contains various gene products but not for specific genes. However, we intended to have an exact mapping between the ontology and both datasets. So we chose the Ontology of Genes and Genomes (OGG) instead, which consists of 69,688 specific genes in different organisms [5]. In addition, we chose the Human Disease Ontology (DOID) for disease entities, consisting of 18,055 terms for various human diseases [3]. Both ontologies are well-established in the biomedical community and suitable for our task.

### 2.2 Generating ontology embeddings

OWL2Vec* [13] provides options for multi-ontology embeddings. However, the problem in our case was that OGG does not have any overlapping with DOID. Thus, the embedding method will not have any natural solution for the spatial alignment of the ontologies inside the embedding space but only concatenate two ontologies. In our approach, we chose two levels of annotation quantities from the TGBA dataset for the number of associations appearing. We selected the 45 most mentioned associations based on the first threshold and added edges between the respective terms from two ontologies to bridge them.

Furthermore, we added 75 more bridges based on the second threshold and made the total number 120. Because of the complicated structure of both enormous ontologies, annotating this small number of bridges was already highly time-consuming. In addition, note that the terms occurring in the datasets comprise only a minor subset of the terms DOID and OGG. However, we decided not to neglect those not occurring in the datasets and embedded the whole ontology in both cases. Although doing that may reduce the time to train the embedding model and potentially influence the model performance, we intended to keep this human annotation part's simplicity and explore some generality capabilities for further research using different ontologies.

### 2.3 Models

We have employed pre-trained BERT (Bidirectional Encoder Representations from Transformers) [15] as our baseline for the classification task, which is a pre-trained neural network using multi Transformer [14] encoder blocks.

ERNIE [16], a knowledge-enriched Masked Language Modelling (MLM), infuses a knowledge graph as additional knowledge to the BERT model using pre-annotated informative entities in every sample. In our case, these informative entities are pre-annotated gene and disease entities. We chose BERT over BioBERT [19], as the latter model should be more suitable for biomedical tasks, but the ERNIE pre-training was initialized with pre-trained BERT weights [16]. Hence, to be able to fairly compare baseline results with ERNIE we decided to employ BERT for baseline rather than the, possibly advantageous, BioBERT.

### 2.4 Another approach: KGE on multiple ontologies

We have also tried using various state-of-the-art KGE methods to embed our ontologies to further increase the generalizability of this attempt and reduce the complexity of the manual annotation part. Notice OWL2Vec* takes the ".owl" file as input [13]. Such file types can be only modified by some specific tools like "Protégé" [20]. In contrast, some KGE packages can take only a ".csv" file consisting of triples, i.e., (h,r,t), while head and tails are entities, and r is their relation.

In our case, the relation consists of three types: two intern hierarchy relations ("*SubclassOf*") in both ontologies and the bridges between two ontologies. The "*SubclassOf*" relation consists of 80,788 samples, and the "Associated" relation consists of 54,308 (27,154) samples. Notice that we need to double the number of relations as the associations between gene and disease are bidirectional, and this fact is crucial in knowledge graph embedding.

## 3 Results

### 3.1 Gene-disease association prediction

We adopted 128 as our input sequence length for both datasets. Although TBGA has a slightly longer average length, the model performed worse when using 256 as the input length. Besides, the prompts we derived from DISGENET are short sentences.

We explored the model performance on three different data inputs, i.e., original TBGA datasets with four classes, TBGA with only two classes, whether gene and disease are associated, and DisGeNet with the same two classes. The test accuracy on each dataset using different embeddings is listed in Table 1. The ERNIE model performed slightly better using the same experiment setup (Supplementary Table 2) than BERT. As the number of bridges increases, the results demonstrate a slightly increasing trend. The embedding of OGG and DOID with 120 bridges from TBGA had the highest accuracy score among the two TBGA datasets. Although the improvement in overall performance is slight, considering that we have only a limited number of manual bridges, this result is still positive.

**Table 1:** Performance predicting gene-disease associations across models: In this table, we show multiple experiment results on two different datasets with different ontology embedding (OE) as input. The “None” embedding denotes the baseline of the natural language classification task performed by BERT. “Ontologies split by comma” denotes that we use the multi-ontology embedding approaches from OWL2Vec* [13] directly. All the other models were trained on ERNIE [16]. Bold represents the best result of each dataset.

Dataset	OE	Test Acc
TBGA(4 classes)	None	69.23 %
TBGA(4 classes)	OGG, DOID	69.42 %
TBGA(4 classes)	OGG + DOID(45 bridges)	69.64 %
TBGA(4 classes)	OGG + DOID(120 bridges)	**72.92 %**
TBGA(2 classes)	None(BERT)	78.12 %
TBGA(2 classes)	OGG, DOID	78.63 %
TBGA(2 classes)	OGG + DOID(45 bridges)	78.01 %
TBGA(2 classes)	OGG + DOID(120 bridges)	**79.02 %**
DisGeNET	None	**77.17 %**
DisGeNET	OGG, DOID	75.2 %
DisGeNET	OGG + DOID(45 bridges)	75.96 %
DisGeNET	OGG + DOID(120 bridges)	76.83 %

**Table 2:** Results for link prediction with some mainstream KGE methods.

KGE	MR	Hits@		
		1	3	5
SimplE [21]	0.2319	0.1917	0.2827	0.4389
UM [22]	0.1033	0.0001	0.3834	0.4823
KG2E [23]	0.0903	0.1950	0.3283	0.4021
ComplEX [24]	0.1967	0.7171	0.7321	0.7335
RotatE [25]	0.0666	0.5402	0.6254	0.6556
PairRE [26]	0.0593	0.6887	0.7228	0.7309
TorusE [27]	**0.1166**	**0.7251**	**0.7420**	**0.7460**

For the DisGeNET dataset, BERT has achieved a slightly better result. Unfortunately, the prior knowledge using TBGA associations did not generalize on DisGeNet, but more bridges still showed a trend of improvement.

### 3.2 Link prediction

The given triples are extracted from the TBGA training and test set, the NA relations are now neglected. Link prediction approach aims to predict more gene-disease associations, which were not covered by the TBGA dataset.

Table 2 shows the experiment results using multiple different KGEMs. We have considered Mean Rank(MR) and Hits@ as evaluation metrics. TorusE [27], a recent work improved TransE [6] via changing the embedding space achieved very promising results in some benchmark dataset of link prediction, performed best for our dataset. Other translational-based KGE like PaiRE [26], RotatE [25] had also achieved competitive results. Bilinear models like ComplEX [24] also achieved promising results, particularly great performance on the Hits@1 score, which is crucial for association prediction.

## 4 Conclusion

In this paper, we explored the power of using the embedding of two ontologies as prior knowledge by evaluating a specific task, i.e., gene and disease association prediction.

The experiment results show that adding a small amount of "bridges" between ontologies increased the model's performance. Our work shows that allowing language models to leverage structured knowledge from ontologies does come with clear advantages in the performance. Besides, the annotation stage brought out in this paper is constrained in reasonable complexity.

Unfortunately, this approach did not generalize well on other datasets. Nevertheless, adding more prior knowledge can further improve the performance, as this trend has already been shown in the main result. The question remains for future researchers if there is a threshold for the number of associations when enough prior knowledge is given, whether the model's generalizability will increase further for this specific task, or it will become saturated at a certain point.

Meanwhile, all experiments are only fine-tuning on some pre-trained models. A well-designed pre-training strategy that adopts biomedical texts for ERNIE, similar to BioBERT [19], may significantly increase the performance on these kinds of tasks. The same concept can also be applied to different ontologies for different kinds of association prediction. In addition, some state-of-the-art KGE has also achieved good results in the link prediction approach, mainly the competitive result of the Hits@1 score.

In conclusion, our work demonstrates the benefits of utilizing structured knowledge from ontologies as prior knowledge in natural language processing tasks, e.g., gene-disease association prediction, along with the potential to improve furthermore in future works.

## Data availability statement

Data associated with this study has been deposited at https://github.com/Yihao21/MultiOE-4-GDA-Prediction.

## Additional information

Supplementary content related to this article has been published online at https://doi.org/10.1016/j.heliyon.2023.e21502.

## CRediT authorship contribution statement

**Yihao Wang:** Conceptualization, Formal analysis, Software, Validation, Writing – original draft, Investigation. **Philipp Wegner:** Conceptualization, Formal analysis, Writing – review & editing, Investigation. **Daniel Domingo-Fernández:** Formal analysis, Investigation, Supervision, Writing – review & editing. **Alpha Tom Kodamullil:** Formal analysis, Project administration, Supervision.

## Declaration of competing interest

The authors declare the following financial interests/personal relationships which may be considered as potential competing interests:Tom Kodamullil reports article publishing charges was provided by Fraunhofer Institute for Algorithms and Scientific Computing SCAI Department of Bioinformatics.
