# Supplementary material for: Multi-ontology embeddings approach on human-aligned multi-ontologies representation for gene-disease associations prediction
Source: Heliyon. 2023 Oct 30;9(11):e21502. doi: 10.1016/j.heliyon.2023.e21502 (PMC10651438; doi:10.1016/j.heliyon.2023.e21502)
Supplement: Multimedia component 1 [file mmc1.docx]

# **Supplementary File**

# **Supplementary Tables**

**Supplementary Table 1. Number of samples in the TBGA dataset.**

|  | **NA** | **Theraoeutic** | **Biomarker** | **Genomic alternative** | **Sum** |
| --- | --- | --- | --- | --- | --- |
| **Training set** | 29,838 (53.4%) | 1,382 (2.4%) | 9808 (17.2%) | 15,963 (28%) | 56,992 |
| **Balanced**  **Sub training set** | 13,116 (35.21%) | 1,375 (3.69%) | 9,751 (26.18%) | 13,007 (34.92%) | 37,249 |
| **Validation set** | 3,874 (58.81%) | 216 (3.28%) | 1,358 (20.62%) | 1,139 (17.29%) | 6,587 |
| **Test set** | 3,833 (58.55%) | 208 (3.18%) | 1,423 (21.74%) | 1,083 (16.54%) | 6,547 |

**Supplementary Table 2. Experiment setting: Hyperparameters choice.**

| **Parameter** | **Value** |
| --- | --- |
| optimizer | AdamW |
| base learning rate | 1e-5 |
| batch_size | 16 |
| input_length | 128 |
| warmup_steps | 100 |
| epochs for fine tuning | 3 |
